# Artificial Intelligence for Mental Health Monitoring: A Solution for Digital Behavioral Health Care and Education—An Umbrella Review

**DOI:** 10.1002/hsr2.71703

**Published:** 2025-12-29

**Authors:** Sumaiya Yeasmin, Mst Masuma Akter Semi, Moustaq Karim Khan Rony, Srabani Das, Anseena Anees Sabeena, Rukshanda Rahman, Barna Biswas, Fahad Ahmed, Adib Hossain

**Affiliations:** ^1^ Department of Psychology St. Francis College Brooklyn New York USA; ^2^ Department of Education Westcliff University Irvine California USA; ^3^ Miyan Research Institute International University of Business Agriculture and Technology Dhaka Bangladesh; ^4^ Department of Business Administration Westcliff University Irvine California USA; ^5^ Department of Computer Science Westcliff University Irvine California USA; ^6^ Department of Technology & Engineering Westcliff University Irvine California USA; ^7^ Department of Science Trine University Angola Indiana USA; ^8^ Department of Business Analytics Trine University Angola Indiana USA

**Keywords:** artificial intelligence, digital behavioral health, health technologies, mental health monitoring, umbrella review

## Abstract

**Background:**

The global burden of mental health disorders continues to escalate, placing immense strain on healthcare systems already challenged by workforce shortages and systemic barriers. As traditional models struggle to meet rising demands, artificial intelligence (AI) has emerged as a promising tool for enhancing the detection and monitoring of psychological distress.

**Aim:**

This umbrella review aimed to synthesize high‐level evidence on how AI is employed in mental health monitoring and assess its potential to support digital behavioral health care and education.

**Methods:**

Following PRISMA 2020 guidelines, the review integrated findings from 29 systematic reviews, scoping reviews, and meta‐analyses published between 2013 and 2025. Studies were selected using a rigorous, multistage screening process and evaluated through the Joanna Briggs Institute appraisal tools. Thematic synthesis was applied to extract and organize recurring insights from diverse digital and clinical contexts.

**Results:**

The results revealed that AI technologies, including machine learning, natural language processing, wearable sensors, and chatbots, enhance diagnostic accuracy, predict crises, and improve access to care. AI's adaptability across mobile platforms, educational settings, and telehealth environments was particularly evident, showing promise for underserved and stigmatized populations. However, concerns around data privacy, algorithmic bias, and user trust were recurrent themes that demand ethical safeguards and transparent governance.

**Conclusion:**

AI is redefining how mental health care is delivered by enabling proactive, personalized interventions. While challenges remain, the responsible and inclusive deployment of AI offers a transformative pathway toward accessible, real‐time mental health support that bridges gaps in traditional care systems.

## 1 Introduction

Mental health has become a critical concern in the global health landscape. The rising prevalence of psychological disorders, from mood instability to severe emotional distress, has placed a significant burden on healthcare systems worldwide [1]. These challenges not only affect individual well‐being but also lead to widespread social and economic consequences [2]. The situation is further worsened by a global shortage of trained mental health professionals, especially in underserved and economically disadvantaged areas [3]. Even in more developed healthcare infrastructures, individuals often encounter barriers such as social stigma, financial limitations, long waiting times, and inconsistent care [4]. As mental health issues increase across all age groups, particularly among younger populations [5], there is an urgent need for innovative approaches that enable early detection, ongoing monitoring, and timely intervention for individuals in psychological distress [6].

Digital health technologies have emerged as practical complements to conventional mental health services [7]. These include mobile health (mHealth), telepsychiatry, wearable biosensors, and digital therapeutic platforms, which extend care beyond clinical settings [8, 9]. Among these, artificial intelligence (AI) has gained attention for its capacity to deliver personalized, real‐time mental health support [10, 11]. AI's ability to process vast data sets, recognize subtle patterns, and generate predictive insights positions it as a transformative tool in behavioral health [12, 13, 14]. When embedded into digital platforms, AI systems can continuously track behavioral, physiological, emotional, and contextual signals to identify early signs of distress before they escalate [15, 16]. This capability is particularly valuable for populations such as students, remote workers, or individuals in marginalized communities who may not have regular access to in‐person care [17].

AI technologies for mental health monitoring operate through both passive and active data collection strategies [18]. Passive monitoring involves the analysis of background data such as keystroke dynamics, sleep patterns, social media behavior, and voice tone to infer psychological states [19]. Active monitoring uses direct interactions with digital agents or assessments to gather self‐reported symptoms and cognitive indicators [20]. Natural language processing (NLP), emotion recognition, and sentiment analysis have proven effective in identifying linguistic and vocal cues linked to distress, mood changes, or suicidal ideation [21, 22]. AI chatbots have shown early success in delivering cognitive behavioral interventions [23, 24], and wearable sensors equipped with predictive algorithms can detect panic attacks or depressive episodes based on physiological changes [25]. These diverse applications indicate that AI can support both early diagnosis and long‐term mental health tracking across a range of populations [26, 27, 28].

Despite its promise, AI integration into mental health care presents several challenges. Ethical concerns such as data privacy, surveillance, informed consent, and algorithmic transparency are increasingly prominent [29, 30]. Many AI systems rely on training data sets that lack demographic diversity, which can result in biased outputs and reduce their effectiveness in varied populations [31]. The complexity and variation among AI models also complicate efforts to standardize metrics for evaluating accuracy, clinical relevance, and effectiveness [32, 33]. These difficulties are compounded by a fragmented body of literature, where studies vary widely in scope, methodology, and reporting quality, making it difficult to draw definitive conclusions. In light of this, a comprehensive umbrella review is needed to synthesize high‐level evidence on AI's role in mental health monitoring, and to assess its implications for sustainable digital behavioral health care and education.

## 2 Methodology

### 2.1 Review Design and Framework

**Figure 1:**
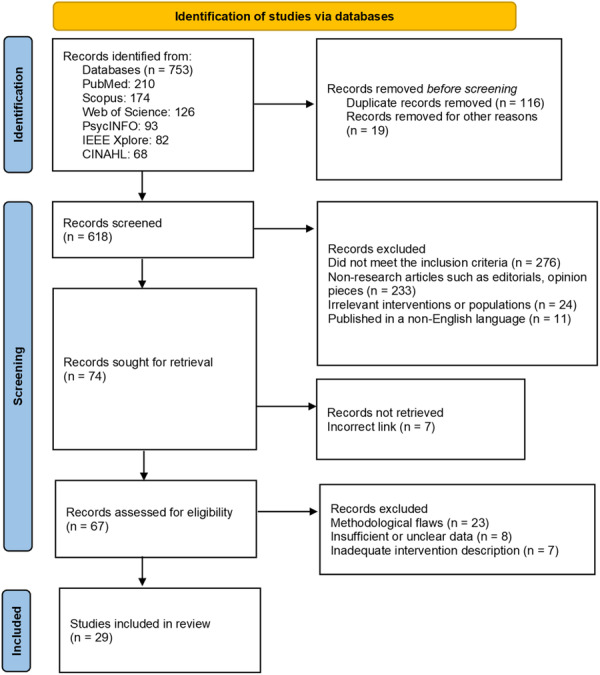
PRISMA flow diagram.

This umbrella review was developed in alignment with the PRISMA 2020 guidelines [34] (Figure 1) to ensure systematic rigor, transparency, and reproducibility throughout the research process. Designed as a comprehensive synthesis of high‐level evidence, the review integrated findings from previously published systematic reviews, scoping reviews, and meta‐analyses to explore the use of AI in mental health monitoring. The focus extended across diverse contexts, including clinical, educational, and digital health environments. The review protocol was outlined prior to data collection to minimize bias and maintain methodological integrity. Structured around a well‐defined research question, the review employed a detailed search strategy, multiphase selection and screening process, methodological quality appraisal using the Joanna Briggs Institute (JBI) tools, and thematic synthesis of key findings. This design enabled the consolidation of broad, cross‐disciplinary evidence into an integrated understanding of AI's roles and implications in digital mental health.

### 2.2 Research Question

Guided by a central question, this review sought to understand: “How is artificial intelligence utilized in monitoring mental health, and what are its implications for digital behavioral health care and education?” This question was structured using the PICO framework, a tool that guided the review's inclusion logic. The population included individuals of all age groups, from children to older adults, who were either experiencing or at risk for mental health issues. The intervention focused on AI‐driven monitoring technologies, including predictive analytics, wearable devices, NLP systems, and conversational agents. No specific comparison was required due to the nature of the included reviews, many of which involved observational or descriptive synthesis. The outcomes of interest were centered on the effectiveness, feasibility, scalability, and practical challenges associated with implementing AI tools for mental health monitoring across healthcare and educational settings.

### 2.3 Eligibility Criteria

To ensure the relevance and quality of included evidence, a strict set of eligibility criteria was applied. This umbrella review considered peer‐reviewed articles that were themselves systematic reviews, scoping reviews, or meta‐analyses. Only studies published in English between January 2013 and February 2025 were included, capturing over a decade of technological and clinical developments. Eligible reviews had to focus on AI technologies used to monitor, assess, or predict mental health or emotional well‐being. The included studies had to involve populations of any age group, regardless of geographic setting, and could be situated in clinical institutions, schools, telehealth platforms, or community‐based mental health services. Primary studies, conference abstracts, opinion pieces, and narrative reviews were excluded to maintain a high level of evidence quality. Notably, reviews focusing exclusively on generative AI models or large language models (LLMs) without a monitoring or assessment component were excluded to preserve thematic focus. Additionally, only studies that provided explicit information about AI methodologies and their application in mental health contexts were considered.

### 2.4 Search Strategy

A thorough search strategy was developed to identify relevant reviews across multiple electronic databases. The search was conducted across PubMed, Scopus, Web of Science, PsycINFO, IEEE Xplore, and CINAHL, ensuring interdisciplinary coverage from health sciences, psychology, and computer science. Both controlled vocabulary (e.g., MeSH terms) and free‐text keywords were employed to maximize retrieval. The search terms included combinations such as “Artificial Intelligence,” “AI,” “machine learning,” “deep learning,” and “natural language processing” alongside mental health‐related terms like “psychological well‐being,” “emotional distress,” and “mental health monitoring.” These were combined with context‐specific terms like “digital health,” “telehealth,” “mHealth,” and “education” using Boolean operators (AND, OR) to structure the query. All search results were exported to the Rayyan screening platform, where duplicates were removed, and screening commenced.

### 2.5 Study Selection Process

The selection process was carried out in two distinct phases: initial screening and full‐text review. In the first phase, titles and abstracts were independently screened by two reviewers to determine potential eligibility. Any disagreements or uncertainties at this stage were resolved through discussion or by consulting a third reviewer. Following this, full‐text versions of the shortlisted articles were retrieved and assessed against the inclusion criteria. During this phase, an emphasis was placed on ensuring that the studies were true systematic or scoping reviews, including mental health as a primary focus, and featured the use of AI‐based monitoring tools. Of the 753 records identified through the database search, 116 duplicates were removed. From the remaining articles, 67 were evaluated at the full‐text stage, and 29 reviews met the final inclusion criteria. The selection process was documented using a PRISMA flow diagram to maintain transparency.

### 2.6 Data Extraction

Data extraction was conducted using a standardized template designed to capture relevant characteristics of the included reviews. The form was piloted on a subset of articles to ensure clarity and reliability. The extracted information included the authors' names and year of publication, the number of included studies, geographic focus, type of review, population characteristics, specific AI technologies employed, outcome domains, and the key conclusions drawn by each review (Table 1). Particular attention was paid to the methodologies used within the included reviews, the robustness of their findings, and how AI tools were integrated into the mental health monitoring process. The extracted data were first organized in Microsoft Excel, which served as a working database for initial categorization. Subsequently, these data were imported into NVivo 14 software to facilitate in‐depth thematic coding and analysis.

**Table 1:** Summary of reviewed studies on the application of artificial intelligence in mental health.

References	Authors and year of publication	Number of included studies	Geographic focus	Type of review	Population characteristics	AI technologies employed	Outcome domains	Key conclusions
[35]	Park et al. (2024)	95	Global	Systematic review	Individuals with depressive disorders (no age restrictions)	CNN, SVM, RF, Gradient Boosting, Decision Tree, Logistic Regression, RNN, Elastic Net, Gaussian Processes, BPNN	Prediction, diagnosis, treatment, monitoring, disease progression, and prescription prediction of depressive disorders	AI demonstrates high accuracy in predicting and managing depressive disorders, especially when using CNN with biomarker or EEG data. Digital healthcare enables non‐face‐to‐face and personalized treatment, offering scalable solutions to address mental health care shortages and enhance accessibility.
[36]	Dehbozorgi et al. (2025)	15	Global	Systematic review	General population including youth, elderly, college students, and global app users	Chatbots (e.g., Wysa, SimSimi), CNN, LSTM, ANN, structural equation modeling, wearable tech integration, and facial recognition AI	Early detection, diagnosis, emotional recognition, symptom monitoring, user engagement, personalized support, psychological assessment	AI applications in mental health demonstrate effectiveness across varied settings, offering personalized and scalable interventions. However, ethical challenges such as data privacy and methodological limitations need to be addressed for sustainable integration into clinical practice.
[37]	Thieme et al. (2020)	54	Global (predominantly developed countries)	Systematic review	Individuals with psychosocial mental health conditions (e.g., depression, anxiety, PTSD, bipolar disorder), healthy individuals, students, older adults, and clinical patients	Machine Learning techniques including supervised learning, unsupervised learning, clustering, CNNs, RNNs, Bayesian models, ensemble methods, NLP techniques, and multimodal ML systems	Detection, diagnosis, symptom monitoring, risk prediction (e.g., suicide), understanding mental health behaviors, treatment improvement, clinician–patient communication, personalization of interventions	ML in mental health has great potential for early detection, diagnosis, and treatment personalization, yet is challenged by ethical concerns, data bias, generalizability, and lack of real‐world deployment. Stronger interdisciplinary and human‐centered approaches are needed for implementable solutions.
[38]	Casu et al. (2024)	15	Global	Scoping review	General population; adolescents, young adults, older adults, individuals with depression, anxiety, panic disorder, Parkinson's disease, substance use disorders, and cancer survivors	AI chatbots utilizing NLP, ML, DL, rule‐based systems, sentiment analysis, reinforcement learning, and user feedback loops; includes LLMs like GPT	Mental health symptom reduction (depression, anxiety, panic, substance use); prevention (eating disorders, HIV); emotional well‐being; adherence to therapy; usability and engagement	AI chatbots show promise in improving mental health and emotional well‐being, offering accessible, scalable support. Effectiveness varies by context and user engagement. Usability and ethical concerns (e.g., privacy, relational bonding) remain challenges. Integration into healthcare systems and personalization are key future priorities.
[39]	Yaacob et al. (2023)	39	Global	Systematic review	General adult populations in tasks such as driving, simulated work, pilots, and sleep research; healthy individuals	SVM, CNN, LSTM, RNN, DNN, ESTCNN, MHLCNN, DSCNN, Bayesian CNN, GDANN, KNN, RF, LightFD Tree, DASL, PSO‐H‐ELM, NU Learning, FLDA, QDA, Fuzzy Neural Networks (RSEFNN, SONFIN, TRFN)	EEG‐based mental fatigue detection, signal acquisition, preprocessing, feature extraction, classification, model performance	AI techniques, especially CNN and SVM, can accurately detect mental fatigue using EEG‐BCI systems. Challenges include data scarcity, imbalance, lack of real‐world validation, and ethical concerns. Future research should focus on XAI, multimodal data, adaptive analysis, and hardware implementation.
[40]	Cruz‐Gonzalez et al. (2025)	85	Global	Systematic review	Individuals with mental health conditions including depression, anxiety, PTSD, schizophrenia, bipolar disorder, OCD, postpartum depression, suicidal ideation, and healthy controls	Support Vector Machine, Random Forest, Logistic Regression, CNN, RNN, Decision Trees, Naive Bayes, Elastic Net, LASSO, NLP, Deep Learning, Ensemble methods, Bayesian Networks	Diagnosis, symptom monitoring, treatment effectiveness, risk prediction (e.g., suicide), intervention efficacy, quality of life assessment	AI shows promise in accurately diagnosing, monitoring, and intervening in mental health conditions. SVM and RF are widely used for diagnosis, while AI chatbots and machine learning models are effective for interventions and monitoring. Challenges include data quality, ethical concerns, and generalizability.
[41]	Zhang et al. (2021)	74	Global	Scoping review	Individuals with neurological and psychiatric conditions including Alzheimer's disease (AD), Parkinson's disease (PD), major depressive disorder (MDD), schizophrenia (SCZ), attention‐deficit/hyperactivity disorder (ADHD), and autism spectrum disorder (ASD)	Support Vector Machine (SVM), Artificial Neural Networks (ANN), Random Forest (RF), Gradient Boosting Decision Trees (GBDT), Convolutional Neural Networks (CNN), Generative Adversarial Networks (GAN), Recurrent Neural Networks (RNN), Deep Belief Networks (DBN), Deep Boltzmann Machines (DBM), Transfer Learning	Classification and diagnosis of neurological and psychiatric diseases using MRI data; evaluation of model accuracy, efficiency, and applicability to clinical practice	AI technologies, especially ML and DL models, have shown promising results in MRI‐based classification of brain disorders. SVM, CNN, and deep models are highly effective. Transfer learning and 3D‐CNN models enhance performance, but data limitations, generalizability, and clinical integration remain challenges.
[42]	Edavally et al. (2021)	13	Global	Systematic review	Individuals with mood disorders (e.g., major depressive disorder, bipolar disorder) and those at risk of suicide	Machine Learning, Random Forest, Support Vector Machine, Artificial Neural Networks, Logistic Regression, Decision Trees, Hybrid Models, Ensemble Methods	Diagnosis of mood disorders, prediction of suicide risk, symptom classification, biomarker identification, risk stratification	AI techniques, particularly ML, RF, and SVM, show promise in diagnosing mood disorders and identifying suicide risk. However, limited generalizability, small sample sizes, and lack of standardized validation methods are key challenges. Further research is needed for broader implementation.
[43]	Zidaru et al. (2021)	144	Global (noted emphasis on UK, USA, Australia, the Netherlands)	Scoping review	Patients and public groups engaged in mental health care design or delivery; includes underserved, indigenous, culturally diverse, youth, and older adults	Machine Learning, Natural Language Processing, Sentiment Analysis, Chatbots, mHealth, Wearables, iHealth, Deep Learning	Applications in assessment, diagnosis, therapy, integration/personalization of care; ethics of public engagement; public involvement in AI planning, development, implementation, and diffusion	AI technologies show promise in enhancing mental health care delivery and patient outcomes. Effective public and patient involvement (PPI) is critical to ensure ethical, inclusive, and contextually appropriate design and use of AI. Future development must integrate principles of design justice and prioritize patient empowerment, trust, and engagement.
[44]	Xian et al. (2024)	144	Global (notable contributions from USA, China, India, UK, Korea)	Scoping review	General public, individuals with depression, anxiety, bipolar disorder, PTSD, eating disorders, schizophrenia; students, clinicians, peer supporters, suicide gatekeepers	Advanced Generative AI (e.g., GPT‐2, GPT‐3, GPT‐4, LSTM, GAN, DialoGPT, HyperCLOVA, PanGu, Midjourney)	Detection, counseling support, therapeutic application, clinical training, clinical decision‐making, goal‐driven optimization	Advanced GAI shows potential in enhancing mental health care through diverse applications, especially in therapy and counseling. However, limitations in diagnostic accuracy, ethical risks, privacy concerns, and technical constraints underscore the need for cautious integration and human‐centered design.
[45]	Abd‐Alrazaq et al. (2020)	12	Global (USA, UK, Sweden, Japan, Australia, Turkey, China, etc.)	Systematic review and meta‐analysis	Individuals with depression, anxiety, acrophobia, and general psychological distress; both clinical and nonclinical samples	Chatbots (rule‐based and AI‐driven) with NLP, ML capabilities; implemented via standalone software or web‐based platforms	Depression, anxiety, psychological well‐being, positive/negative affect, distress, stress, acrophobia, and safety	Chatbots may improve mental health outcomes such as depression, distress, stress, and acrophobia, but evidence is weak and inconsistent. More rigorous RCTs with larger sample sizes are needed to assess their clinical effectiveness and safety.
[46]	Thenral and Annamalai (2020)	253	India (telepsychiatry), Global (AI in mental health)	Scoping review	Individuals with mental health conditions in India and globally; includes COVID‐19‐affected populations	AI and ML technologies including web‐based platforms, social media integration, internet games, asynchronous models, EHR analysis, chatbots, CDSS, AR/VR, NLP	Clinical decision support, diagnosis, personalized medicine, health‐seeking behavior, data‐informed psychiatric care, remote consultations	Telepsychiatry and AI hold promise for addressing mental health needs in India, especially post‐COVID. Indigenous tech development, validation, and ethical considerations are essential. Effective integration requires psychiatrist–technologist collaboration and public engagement.
[47]	Welch et al. (2022)	19	Global (most studies conducted in the USA)	Scoping review	Children and adolescents (0–18 years) with psychiatric disorders including ASD, ADHD, and internalizing disorders	Wearable AI devices including wrist‐worn biosensors, ECG chest straps, accelerometers, gyroscopes, EEG headsets; feature extraction and machine learning approaches for diagnosis and behavior prediction	Diagnosis, treatment evaluation, behavioral prediction, emotional recognition, cognitive engagement monitoring, and physiological stress detection	Wearable AI technologies show promise in diagnosing and predicting behavioral symptoms in child psychiatry, especially for ASD and ADHD. There is a lack of randomized controlled trials and small sample sizes limit generalizability. Integration into real‐world settings and broader diagnostic categories is needed.
[48]	Abd‐Alrazaq et al. (2023)	69	Global (including USA, Mexico, UK, Norway, Japan, South Korea, etc.)	Scoping review	Primarily adults aged 18–65 with anxiety or depression; some studies included children (< 18) and older adults (≥ 65)	Machine Learning (e.g., Random Forest, SVM, Logistic Regression, Decision Tree, XGBoost, KNN, AdaBoost); Deep Learning (e.g., CNN, MLP)	Diagnosis, screening, monitoring, prediction of anxiety and depression using wearable data (e.g., activity, sleep, heart rate, mental health measures)	Wearable AI shows promise for diagnosing and monitoring anxiety and depression. Most studies focused on diagnosis; none explored treatment. Physical activity, sleep, and heart rate were the most common data sources. Future work should focus on treatment applications, data quality, ethical concerns, and combining wearable data with self‐reported inputs for accuracy.
[49]	Abd‐Alrazaq et al. (2023)	21	Global (USA, UK, Pakistan, Japan, China, Germany, Hong Kong, Lithuania, Mexico, Taiwan)	Systematic review and meta‐analysis	Adults with anxiety‐related disorders, including generalized anxiety, social anxiety, panic disorder, and specific phobias; average age ~35 years	SVM, RF, Decision Tree, KNN, MLP, Logistic Regression, LSTM, XGBoost, CNN, Gradient Boosting, Ensemble models, LDA	Detection and prediction of anxiety using wearable data (heart rate, sleep, activity, EEG, skin temperature, EDA, etc.); model performance (accuracy, sensitivity, specificity)	Wearable AI shows good performance (accuracy = 82%, sensitivity = 79%, specificity = 92%) but is not yet ready for clinical use. Future research should improve model robustness, test across broader wearables, include neuroimaging data, and differentiate types of anxiety.
[50]	Auf et al. (2025)	12	Global (predominantly high‐income countries including USA, Canada, UK, China)	Scoping review	Individuals with various mental health issues including depression, substance use disorder, autism, suicide risk; healthcare professionals and patients in decision‐making roles	AI models including ML, DL, NLP; systems categorized as diagnostic and predictive AI, treatment selection AI, and self‐help AI (e.g., chatbots)	Support in diagnosis, treatment selection, self‐care, shared decision‐making, physician–patient communication, AI explainability, and trust	AI systems show promise in supporting decision‐making in mental health care, but integration faces challenges including accuracy, trust, engagement, and workflow disruption. Few systems support shared decision‐making explicitly. More empirical studies are needed to assess long‐term integration and real‐world effectiveness.
[51]	Jin et al. (2025)	95	Global (notably USA, China, Canada, India, Israel, UK, Germany, Iran, etc.)	Scoping review	Individuals with depression, anxiety, PTSD, schizophrenia, autism, bipolar disorder, OCD, BPD, ADHD, suicidal ideation; clinicians, peer supporters, general public	Large Language Models (e.g., GPT‐3.5/4, BERT, RoBERTa, LLaMA, Bard, Claude, PsychBERT, MentalRoBERTa, ChatGPT, Fine‐tuned LLMs)	Screening/detection of mental disorders, clinical treatment support, suicide risk prediction, mental health education, counseling support, chatbot development, emotional assessment, sentiment analysis	LLMs show strong performance in mental health applications, including detection, diagnosis, and intervention. Fine‐tuned LLMs enhance clinical decision‐making and emotional support. Ethical, privacy, and bias concerns must be addressed for real‐world use. Multimodal LLMs and prompt engineering are key future directions.
[52]	Milne‐Ives et al. (2022)	17	Global	Scoping review	Adults with various mental health needs (depression, stress, mood disorders, suicide risk); general public	Random Forest, SVM, Decision Trees, Neural Networks, NLP, Chatbots, LASSO, Bayesian models, Boosting algorithms, PCA	Risk prediction, stress/mood classification, diagnostic support, personalized notifications, conversational support, engagement, and feasibility	AI/ML in mental health apps shows feasibility and diverse applications (e.g., prediction, conversation, personalization), but existing studies are limited in scale, rigor, and duration. Stronger evidence is needed from large‐scale RCTs to evaluate real‐world effectiveness.
[53]	Rogan et al. (2024)	10	Global (notably UK, USA, India, Australia, Germany)	Systematic review with meta‐synthesis	Mental health care professionals (nurses, psychiatrists, psychologists, clinicians, therapists) across diverse care settings	Passive sensing tools (e.g., wearables, mobile apps), AI, ML models for remote monitoring, diagnosis support, behavioral tracking	Clinician views on AI and passive sensing in mental health care: utility, barriers, facilitators, risk perception, data ethics, therapeutic alliance	Clinicians are cautiously open to AI and passive sensing tools but raise concerns about data overload, therapeutic relationships, digital literacy, and privacy. Usability, stakeholder involvement, training, and ethical safeguards are essential for integration into practice.
[54]	Razavi et al. (2024)	98	Global	Scoping review	Individuals with stress and stress‐related mental disorders (e.g., depression, anxiety, PTSD); general population and clinical groups	Support Vector Machines (SVM), Neural Networks (NN, including CNN, RNN, LSTM), Random Forest, Decision Trees, Logistic Regression, Naive Bayes, KNN, Boosting (AdaBoost, XGBoost), Discriminant Analysis, Fuzzy C‐means, K‐means clustering, ensemble methods	Detection, prediction, and monitoring of stress and related disorders; model performance (accuracy, sensitivity); preprocessing techniques and data types (HR, HRV, EEG, questionnaires, etc.)	ML models, particularly SVMs, RF, CNNs, and LSTMs, demonstrate high accuracy in stress‐related disorder prediction. Heart‐related and skin response data are key features. There is a need for real‐time, personalized models, ethical considerations, and interpretability improvements in future research.
[55]	Guo et al. (2024)	40	Global (notably USA, UK, China, multilingual contexts)	Systematic review	Individuals with depression, anxiety, PTSD, suicidal ideation, social anxiety, loneliness; mental health patients and general public	Large Language Models (LLMs) including GPT‐3, GPT‐4, BERT and variants, PsychBERT, DialoGPT, ERNIE Bot, Claude, Bard, ChatGLM, LaMDA	Detection of mental health conditions, suicidal ideation analysis, chatbot intervention, sentiment/emotion classification, treatment planning, diagnosis support, clinical education	LLMs are promising tools for mental health support, showing strong capabilities in detection, support, and diagnosis. However, challenges like hallucinations, lack of clinical judgment, ethical risks, and limited multilingual support hinder real‐world deployment. Ongoing development of specialized data sets, interpretability, and ethical standards is crucial.
[56]	Villarreal‐Zegarra et al. (2024)	21	Global (notably high‐income countries like the USA)	Systematic review and meta‐analysis	Participants of all ages diagnosed with depression or anxiety; includes individuals with mental disorders, chronic diseases, and university students	Natural Language Processing (NLP) models including rule‐based systems and AI‐based NLP (deep learning, machine learning); self‐administered interventions via chatbots, voice/text‐based systems	Reduction of depressive and anxiety symptoms measured through validated scales (e.g., PHQ‐9, GAD‐7); symptom severity, therapy effectiveness	Self‐administered NLP‐based interventions significantly reduce depressive and anxiety symptoms. AI‐based models showed higher effectiveness than controls. Despite promising outcomes, the certainty of evidence remains low due to bias, heterogeneity, and publication bias.
[57]	Rahsepar Meadi et al. (2025)	101	Global	Scoping review	Individuals using conversational AI in mental health care, including those with mental health issues in clinical and nonclinical settings	Conversational AI including psychotherapeutic chatbots (e.g., Woebot, Wysa), AI‐driven virtual therapists using NLP and machine learning	Ethical challenges such as privacy, safety, trust, empathy, accountability, justice, autonomy, effectiveness, employment impact, deception	Conversational AI poses diverse ethical concerns including lack of empathy, risk of harm, privacy violations, and accountability gaps. While CAI increases access to mental health support, its use requires ethical guidelines, human oversight, and further empirical study to ensure responsible deployment.
[58]	Scherbakov et al. (2025)	1768	Global (notably USA, China, UK, India)	Scoping review	Individuals with mental health conditions such as depression, suicide risk, anxiety, PTSD, substance use disorders, schizophrenia, and more	Natural Language Processing (NLP) with methods including CNN, LSTM, RNN, transformers (e.g., BERT, GPT‐3), topic modeling (e.g., LDA), sentiment analysis, rule‐based systems, and neural networks	Detection, classification, sentiment/emotion analysis, prediction of mental health conditions, analysis of social determinants of health, data set sharing and accessibility	NLP plays a major role in mental health research with clinical notes and social media as primary data sources. Depression and suicide are the most studied conditions. SDOH variables are underutilized. Enhanced data set transparency and ethical AI use are essential for reproducibility and equity.
[59]	Bhatt et al. (2022)	37	Global	Scoping review	Individuals experiencing various physical and mental health conditions (e.g., depression, suicidal tendencies, diabetes, sleep apnea, asthma, Parkinson's disease)	Deep Learning (DL), Federated Learning (FL), Explainable AI (XAI), Machine Learning (ML), mobile health (mHealth) sensors, wearable technologies, smartphone‐based AI models	Disease detection and prediction, remote monitoring, suicide prevention, mental health assessment, chronic condition management, physical activity tracking, privacy‐preserving care	AI‐powered mHealth (AIM) is a growing domain supporting disease prevention, chronic condition monitoring, and mental health management. AIM offers personalized care and enhanced remote health services but faces barriers such as lack of public data sets and standardization. FL and XAI can advance adoption while protecting privacy.
[60]	Razavi et al. (2022)	26	Global	Scoping review	Individuals with stress‐related mental disorders; studies used data from both clinical and nonclinical populations through wearable and sensor technologies	Random Forest, Neural Networks (CNN, DRCN), SVM, Decision Tree, Kalman Filter, Genetic Algorithm, Bayesian Classifier, Logistic Regression	Detection and prediction of stress; stress level classification; model accuracy, performance evaluation using physiological data (HR, HRV, skin conductance, respiration, EEG)	Physiological features such as HR, HRV, skin conductance, and respiration are key in stress prediction. Random Forest and Neural Networks are top‐performing models. Future work should consider individual variability and feature coherence for improved generalizability and accuracy.
[61]	Rony et al. (2025)	14	Global	Systematic review and meta‐analysis	Individuals with psychiatric disorders (e.g., depression, schizophrenia, bipolar disorder, ADHD, anxiety, etc.); studies include clinical and nonclinical populations	Machine Learning (e.g., SVM, RF), Deep Learning (e.g., CNN, RNN), and Hybrid Models combining ML and DL with NLP	Diagnostic accuracy and therapeutic efficacy in psychiatric settings; personalized treatment, symptom tracking, risk prediction, therapy optimization	AI demonstrates strong diagnostic accuracy (85%) and therapeutic efficacy (84%) in psychiatry. ML outperforms other models in structured data analysis. Hybrid models show promise in integrating diverse data types. Standardization, ethical safeguards, and inclusive validation are essential for clinical adoption.
[62]	Lee et al. (2025)	5	Global	Scoping review	Medical students, educators, and healthcare professionals involved in psychiatric education	Generative AI (GenAI) tools including ChatGPT‐3.5, ChatGPT‐4, Claude 3, LLaMA 3	Case‐based learning, simulation, content synthesis, and assessment in psychiatric education	GenAI shows promise in enhancing psychiatric education across simulation, case generation, and assessments. Challenges include content accuracy, ethical concerns, and privacy issues. Integration should be guided by clear frameworks, and further research is necessary to optimize and validate its educational impact.
[63]	Abd‐Alrazaq et al. (2023)	54	Global (notably USA, Mexico, South Korea, Norway, Japan, UK)	Systematic review and meta‐analysis	Individuals with depression; some studies included healthy controls, individuals with bipolar disorder, schizophrenia, and mood swings	Random Forest, Logistic Regression, Support Vector Machine, CNN, LSTM, KNN, Gradient Boosting, Ensemble Models, Decision Tree, AdaBoost, Deep Neural Networks, Ridge Regression, etc.	Detection and prediction of depression using wearable data (e.g., activity, sleep, heart rate); model accuracy, sensitivity, specificity, RMSE	Wearable AI demonstrates promising performance (accuracy up to 89%, sensitivity 87%, specificity 93%) in depression detection and prediction, but is not yet ready for clinical use. Further improvements, integration with neuroimaging data, and more robust studies are needed to enhance generalizability and performance.

### 2.7 Quality Appraisal

The methodological quality of the reviews included was critically assessed using the JBI appraisal checklists (Table 2), adapted for different review types [64]. Each included study was evaluated based on criteria such as clarity of the review question, appropriateness of the inclusion criteria, transparency of the search strategy, and methodological rigor in data synthesis. The appraisal process was conducted independently by two reviewers, and any disagreements were resolved through discussion. Studies were not excluded based on appraisal scores alone; however, their quality ratings were considered when interpreting the overall findings. Most reviews were rated as moderate to high in quality, suggesting that their findings could be relied upon with reasonable confidence. The inclusion of multiple high‐quality reviews also provided a layered understanding of the evidence base and enhanced the trustworthiness of the synthesis.

**Table 2:** JBI quality assessment.

References	Authors and year of publication	Review type	Clearly stated review question	Appropriate inclusion criteria	Appropriate search strategy	Adequate sources and resources used	Appropriate criteria for appraising studies	Independent critical appraisal by two or more reviewers	Methods to minimize errors in data extraction	Appropriateness of combining studies	Assessment of publication bias	Recommendations supported by data	Directives for future research provided	Items met (out of 11)	Overall appraisal
[35]	Park et al. (2024)	Systematic review	Yes	Yes	Yes	Yes	Yes	Yes	Yes	Yes	No	Yes	Yes	10	High
[36]	Dehbozorgi et al. (2025)	Systematic review	Yes	Yes	Yes	Yes	Yes	Yes	Yes	Yes	No	Yes	Yes	10	High
[37]	Thieme et al. (2020)	Systematic review	Yes	Yes	Yes	Yes	Yes	Yes	Yes	Yes	No	Yes	Yes	10	High
[38]	Casu et al. (2024)	Scoping review	Yes	Yes	Yes	Yes	Yes	Yes	Yes	Yes	No	Yes	Yes	10	High
[39]	Yaacob et al. (2023)	Systematic review	Yes	Yes	Yes	Yes	Yes	Yes	Yes	Yes	Yes	Yes	Yes	11	High
[40]	Cruz‐Gonzalez et al. (2025)	Systematic review	Yes	Yes	Yes	Yes	Yes	Yes	Yes	Yes	No	Yes	Yes	10	High
[41]	Zhang et al. (2021)	Scoping review	Yes	Yes	Yes	Yes	No	No	No	No	No	Yes	Yes	6	Moderate
[42]	Edavally et al. (2021)	Systematic review	Yes	Yes	Yes	Yes	Yes	Yes	Yes	Yes	No	Yes	Yes	10	High
[43]	Zidaru et al. (2021)	Scoping review	Yes	Yes	Yes	Yes	Yes	Yes	Yes	Yes	No	Yes	Yes	10	High
[44]	Xian et al. (2024)	Scoping review	Yes	Yes	Yes	Yes	Yes	Yes	Yes	Yes	No	Yes	Yes	10	High
[45]	Abd‐Alrazaq et al. (2020)	Systematic review and meta‐analysis	Yes	Yes	Yes	Yes	Yes	Yes	Yes	Yes	Yes	Yes	Yes	11	High
[46]	Thenral and Annamalai (2020)	Scoping review	Yes	Yes	Yes	Yes	No	No	No	No	No	Yes	Yes	6	Moderate
[47]	Welch et al. (2022)	Scoping review	Yes	Yes	Yes	Yes	Yes	Yes	Yes	Yes	No	Yes	Yes	10	High
[48]	Abd‐Alrazaq et al. (2023)	Scoping review	Yes	Yes	Yes	Yes	Yes	Yes	Yes	Yes	Yes	Yes	Yes	11	High
[49]	Abd‐Alrazaq et al. (2023)	Systematic review and meta‐analysis	Yes	Yes	Yes	Yes	Yes	Yes	Yes	Yes	Yes	Yes	Yes	11	High
[50]	Auf et al. (2025)	Scoping review	Yes	Yes	Yes	Yes	Yes	No	No	No	No	Yes	Yes	7	Moderate
[51]	Jin et al. (2025)	Scoping review	Yes	Yes	Yes	Yes	Yes	Yes	Yes	No	No	Yes	Yes	9	High
[52]	Milne‐Ives et al. (2022)	Scoping review	Yes	Yes	Yes	Yes	Yes	No	No	No	No	Yes	Yes	7	Moderate
[53]	Rogan et al. (2024)	Systematic review with meta‐synthesis	Yes	Yes	Yes	Yes	Yes	Yes	Yes	Yes	No	Yes	Yes	10	High
[54]	Razavi et al. (2024)	Scoping review	Yes	Yes	Yes	Yes	Yes	No	No	Yes	No	Yes	Yes	8	Moderate
[55]	Guo et al. (2024)	Systematic review	Yes	Yes	Yes	Yes	Yes	Yes	Yes	Yes	Yes	Yes	Yes	11	High
[56]	Villarreal‐Zegarra et al. (2024)	Systematic review and meta‐analysis	Yes	Yes	Yes	Yes	Yes	Yes	Yes	Yes	Yes	Yes	Yes	11	High
[57]	Rahsepar Meadi et al. (2025)	Scoping review	Yes	Yes	Yes	Yes	Yes	Yes	Yes	No	No	Yes	Yes	9	High
[58]	Scherbakov et al. (2025)	Scoping review	Yes	Yes	Yes	Yes	Yes	Yes	Yes	Yes	No	Yes	Yes	10	High
[59]	Bhatt et al. (2022)	Scoping review	Yes	Yes	Yes	Yes	Yes	No	No	Yes	No	Yes	Yes	8	Moderate
[60]	Razavi et al. (2022)	Scoping review	Yes	Yes	Yes	Yes	Yes	Yes	No	Yes	No	Yes	Yes	9	High
[61]	Rony et al. (2025)	Systematic review and meta‐analysis	Yes	Yes	Yes	Yes	Yes	Yes	Yes	Yes	Yes	Yes	Yes	11	High
[62]	Lee et al. (2025)	Scoping review	Yes	Yes	Yes	Yes	Yes	Yes	Yes	No	No	Yes	Yes	9	High
[63]	Abd‐Alrazaq et al. (2023)	Systematic review and meta‐analysis	Yes	Yes	Yes	Yes	Yes	Yes	Yes	Yes	Yes	Yes	Yes	11	High

### 2.8 Data Synthesis and Analysis

**Figure 2:**
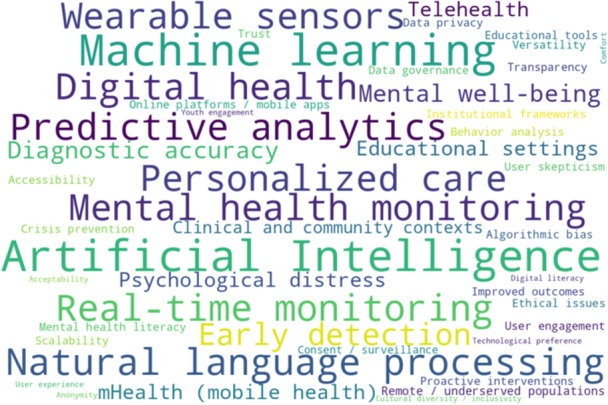
Key terms in AI applications for mental health support.

**Figure 3:**
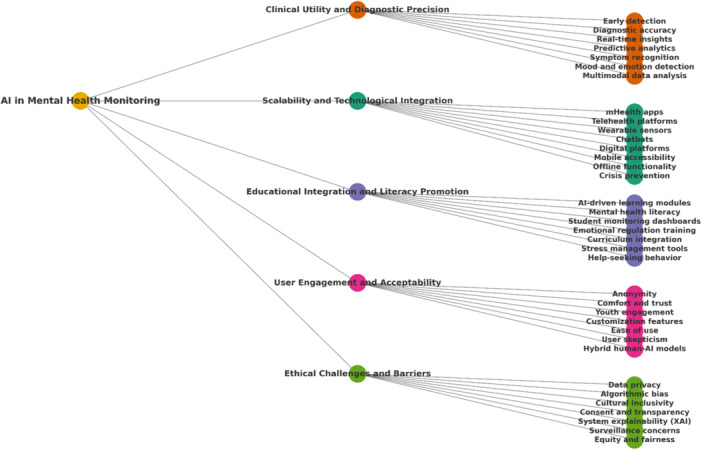
Conceptual framework of artificial intelligence in mental health monitoring.

Given the heterogeneity in population characteristics, types of AI interventions, outcome measures, and study designs, a meta‐analytic approach was not suitable. Instead, a thematic synthesis was adopted to integrate findings across diverse reviews. This approach enabled the identification of recurring patterns, central concepts, and key challenges presented in the literature. The process followed the well‐established six‐step framework proposed by Braun and Clarke. It began with familiarization with the data through repeated reading, followed by the generation of initial codes (Figure 2) that captured key insights related to AI application, functionality, user engagement, and scalability. These codes were then clustered into preliminary themes, which were reviewed and refined to ensure they were coherent and representative of the broader data set. The final themes were clearly named and defined (Figure 3), capturing the nuances of AI integration in mental health monitoring, its practical utility, and barriers to adoption. This thematic approach allowed for a rich, nuanced synthesis that maintained the contextual integrity of the original studies.

## 3 Results

### 3.1 Overview of Studies

This umbrella review analyzed 29 studies [35, 36, 37, 38, 39, 40, 41, 42, 43, 44, 45, 46, 47, 48, 49, 50, 51, 52, 53, 54, 55, 56, 57, 58, 59, 60, 61, 62, 63] exploring AI applications in mental health care, including 9 systematic reviews, 5 with meta‐analysis, 1 with meta‐synthesis, and 14 scoping reviews. These studies covered diverse global settings and populations such as adults, adolescents, and individuals with psychiatric, neurological, or stress‐related conditions. Various AI technologies were used, including machine learning (ML), NLP‐based chatbots, virtual therapists, artificial neural networks (ANN), and augmented or virtual reality (AR/VR). Tools included chatbots, wearable sensors, and diagnostic platforms.

Applications were grouped into diagnostic (e.g., depression detection), predictive (e.g., suicide risk forecasting), and therapeutic (e.g., chatbot‐delivered CBT) categories. Data sources included text (language analysis), audio (voice tone), and physiological signals (heart rate). Deployment contexts ranged from clinical and educational to mhealth environments. Common outcome domains included explainability, assessment tools, MRI‐based diagnosis, model accuracy, and clinician perspectives on passive sensing. Despite AI's promise in enhancing diagnosis, prediction, and therapy, limitations were noted. These include inconsistent methodology, lack of standardization, small sample sizes, and limited external validation. Ethical challenges related to data privacy, transparency, and access equity were also identified. Future research should address these gaps with stronger designs and ethical implementation frameworks.

### 3.2 Enhancing Precision in Mental Health Monitoring

#### 3.2.1 AI‐Driven Diagnostic Accuracy

A prominent finding across the reviewed literature is that AI holds substantial promise in enhancing diagnostic accuracy for mental health conditions when compared to traditional assessment methods. Conventional approaches, such as interviews or self‐reported evaluations, often face limitations including subjectivity, recall bias, and infrequent data collection, which can hinder the timely identification of mental health issues [35]. In contrast, AI systems are capable of continuously observing behavior and interpreting complex emotional patterns, offering more immediate and nuanced insights [40, 43]. Several studies revealed that AI consistently outperformed traditional methods in identifying signs of depression, anxiety, and stress‐related disorders [46, 47]. In one case, the analysis of text and audio inputs showed diagnostic precision exceeding 85%, outperforming conventional evaluations [45]. Other studies demonstrated that the analysis of facial expressions, body language, and vocal features led to even greater accuracy, surpassing 90% in identifying mood and anxiety disorders [49, 63].

Across the reviewed studies, diagnostic accuracy ranged from 78% to 92%, depending on the modality used. Text‐based models averaged 81% to 85% [49, 61], while multimodal systems integrating facial, vocal, and physiological data achieved accuracies above 89% [63]. Moreover, systems that interpreted written and spoken language were notably effective in detecting subtle emotional cues that often go unnoticed in manual assessments [51]. One study that examined communication patterns across digital platforms reported an accurate rate of 88% in identifying depressive symptoms, far exceeding that of traditional clinical evaluations [61]. Comparative analyses in several reviews also indicated that deep learning (DL) architectures (e.g., CNNs, LSTMs) outperformed classical ML models (e.g., SVM, decision trees) by margins of 5–12 percentage points in diagnostic tasks [39]. These findings highlight the growing capability of AI to not only support but, in some cases, surpass human judgment in the early detection and monitoring of mental health conditions.

#### 3.2.2 Predictive and Early Detection Capabilities

AI's predictive and early detection capabilities extend beyond diagnosing current mental health conditions to anticipating future risks and deterioration. This feature is particularly crucial for managing disorders such as suicidal ideation, posttraumatic stress disorder (PTSD), and panic disorders, where timely intervention can be lifesaving [36]. Numerous studies have highlighted the integration of wearable sensors with AI algorithms to identify physiological indicators that preceded anxiety or panic attacks [37, 38]. For instance, one system demonstrated the ability to forecast panic episodes up to an hour in advance by analyzing data such as heart rate variability and skin conductance, offering an unprecedented opportunity for early, targeted responses [48, 55]. In educational settings, AI‐driven monitoring of digital behavior, such as decreased participation in virtual classes or variations in typing patterns, has been effective in detecting early signs of emotional distress [43, 46]. These real‐time insights allow counselors and educators to take proactive measures, leading to notable improvements in student mental health outcomes.

### 3.3 Effective Integration Into Digital Health Platforms

#### 3.3.1 Versatility Across Platforms

AI tools have exhibited remarkable versatility across a range of digital platforms in mental health care. In tele‐mental health environments, AI has been employed to enhance the efficiency of patient intake, prioritize urgent cases, and monitor progress between therapy sessions [50, 58]. NLP‐powered chatbots are frequently embedded within these platforms to offer continuous, on‐demand support that complement traditional counseling [56]. One prominent example involved a mental wellness platform with an AI chatbot capable of holding natural, human‐like conversations [43]. Many users expressed greater comfort discussing sensitive topics with the chatbot, particularly when concerned about stigma or judgment [54]. Importantly, the chatbot was also equipped to escalate serious concerns to human therapists when signs of crisis were detected, thereby safeguarding patient well‐being [35]. Additionally, mHealth applications have leveraged AI for personalized care. These apps, enhanced with sentiment analysis and mood‐tracking capabilities, enabled users to engage in tailored mental health journeys [46]. For example, some apps used ML to assess daily mood entries, sleep habits, and physical activity, generating individualized coping strategies [40, 47]. Research indicates that users of such AI‐enhanced applications experienced more significant reductions in symptoms compared to those using traditional, manual journaling methods [38].

#### 3.3.2 Accessibility and Reach

A key advantage of AI‐based mental health tools lies in their capacity to bridge longstanding gaps in access to care, particularly in rural and underserved regions where mental health professionals are scarce. By leveraging mobile platforms, AI offers an accessible solution that delivers mental health support directly to individuals, regardless of their geographic location [44]. For example, studies involving rural adolescents have shown that AI‐driven mobile applications are regularly used by participants, leading to notable improvements in self‐reported mental well‐being [52]. The app's offline functionality and user‐friendly design facilitated frequent use, even in areas with limited or unstable internet connectivity [37]. Similarly, programs aimed at low‐income populations have harnessed AI to efficiently screen large numbers of individuals, minimizing costs and maximizing reach [47]. Automated triage systems empowered community health workers to identify and prioritize high‐risk cases, enabling better resource allocation and expanding the impact of limited healthcare infrastructure [36].

### 3.4 AI in Educational Settings for Mental Health Literacy

#### 3.4.1 AI‐Driven Educational Tools

Educational institutions are increasingly adopting AI technologies to foster mental health awareness and cultivate emotional intelligence among students. AI‐powered learning modules provide dynamic, personalized educational experiences that align well with the preferences of digitally native learners [39, 42]. These tools often incorporate interactive activities, virtual simulations, and real‐time feedback, making mental health education more engaging and effective [53, 58]. A notable initiative involved a DL‐driven virtual assistant designed to teach stress reduction techniques in both high schools and universities [35]. Students interacted with the assistant via mobile applications and school intranet systems, receiving tailored lessons on emotional regulation, mindfulness, and peer support [44, 52]. The content dynamically adapted to each student's emotional state, enhancing relevance and impact. Postintervention evaluations showed that students exhibited higher levels of mental health literacy and demonstrated a greater willingness to seek professional help, highlighting the potential of AI to promote proactive mental well‐being in educational settings [56].

#### 3.4.2 Integration Within Curriculum and Institutional Frameworks

AI tools have not only served as standalone mental health interventions but have also been seamlessly integrated into the broader academic curriculum and digital infrastructure of educational institutions. Several universities have implemented AI‐enabled dashboards capable of monitoring student well‐being in real time [49]. These systems provide mental health professionals and academic staff with actionable insights derived from behavioral and academic performance data, allowing for prompt and informed interventions [36, 44]. A notable large‐scale deployment involved AI algorithms that tracked patterns in class attendance, assessment performance, and online engagement to identify students potentially experiencing emotional difficulties [45, 53]. When signs of distress were detected, the system initiated automated check‐ins through email or app notifications and referred to high‐risk individuals to student counseling services [51]. This level of integration supports a more holistic and preventive approach to mental health care on campus, helping institutions proactively address student needs before crises emerge [55].

### 3.5 User Experience and Engagement Dynamics

#### 3.5.1 Acceptance and Perceptions of AI

User acceptance emerged as a crucial factor influencing the effectiveness of AI‐driven mental health interventions. Younger users, particularly adolescents and college students, generally expressed favorable perceptions of these technologies [42]. They valued the anonymity, ease of access, and instant responsiveness offered by AI tools, often finding it more comfortable to disclose personal emotions to a chatbot or app interface than in traditional face‐to‐face settings [59]. This was especially true when discussing sensitive topics such as trauma, identity struggles, or substance use [52]. Despite these advantages, some skepticism persisted, especially among older adults, educators, and certain healthcare providers [39, 56]. Their concerns largely revolved around the perceived impersonality of AI interactions, the risk of misinterpreting nuanced emotional cues, and doubts regarding the reliability of algorithm‐generated recommendations [36, 43]. In response to these concerns, several developers introduced hybrid models that combined AI‐driven assessments with human oversight, allowing mental health professionals to review and validate AI‐generated insights before initiating intervention [41, 62].

#### 3.5.2 Barriers and Facilitators

The reviewed studies highlighted a range of barriers and facilitators that influenced user engagement with AI‐based mental health tools. Key facilitators included intuitive, user‐friendly interfaces, options for customization, clearly defined data usage policies, and minimal time requirements [57]. When users felt empowered to personalize the tool's features and understood how their data were being used, their adherence to the intervention and overall satisfaction improved significantly [54, 61]. Conversely, several barriers hindered sustained use. Complicated registration procedures, recurring technical glitches, and ambiguous instructions often discouraged users from continuing with the platform [42, 49]. Among the most frequently reported concerns were issues related to privacy, particularly the use of passive data collection methods involving sensors or social media activity [45]. However, studies indicated that these concerns could be mitigated through transparent communication, including clear explanations about data storage practices, informed consent protocols, and user control settings, ultimately enhancing trust and long‐term engagement [51, 59].

### 3.6 Ethical Implications and Privacy Concerns

#### 3.6.1 Data Security and Confidentiality

Safeguarding user privacy has emerged as a central ethical issue in the deployment of AI technologies for mental health support. Due to the highly personal nature of psychological information, researchers have stressed the importance of implementing strong protective mechanisms [57]. These often include advanced encryption protocols, restricted access based on user roles, anonymizing sensitive data, and secure authentication systems [60]. Such precautions aim to prevent unauthorized use or breaches of confidential information. Nonetheless, user confidence in these systems remains fragile [63]. Incidents involving data leaks or ambiguous privacy terms have fueled apprehension and hesitation [49]. Many users have voiced the need for increased autonomy in managing their personal data [38], suggesting customizable consent settings where individuals can selectively permit monitoring of certain information, such as sleep or physical activity, while declining access to more invasive inputs such as voice or location data [41, 45, 51]. These concerns highlight the urgent need for transparent, user‐first data policies in AI‐driven mental health solutions.

#### 3.6.2 Algorithmic Fairness and Explainability

Algorithmic fairness and explainability have emerged as vital considerations in the responsible use of AI for mental health support. Several studies have shown that AI systems trained on limited or uniform data sets often perform inconsistently when applied to diverse user groups [60, 61]. For instance, emotion recognition tools that were calibrated using data from culturally similar populations tended to struggle when analyzing emotional expressions from individuals with different communication styles or cultural norms [50]. These inconsistencies raise important questions about the inclusivity and accuracy of AI‐generated insights across varied demographic contexts [62]. As a response, the concept of explainable AI (XAI) has gained momentum, emphasizing the need for greater transparency in how AI systems function [63]. Both mental health professionals and users have expressed the need to understand the basis of AI‐generated decisions—particularly when these decisions affect clinical care, educational support, or emergency responses [41]. Systems that offer clear and interpretable outputs, such as visual summaries, behavioral trends, and contextual justifications for alerts, tend to foster higher levels of trust and consistent user engagement [48].

## 4 Discussion

This review brings to light the rapidly evolving role of AI in mental health care, particularly highlighting its capacity to enhance diagnostic accuracy and early detection. Traditional diagnostic tools often rely heavily on subjective interpretations, which may be influenced by factors such as recall bias, social desirability, or infrequent assessments [65]. In contrast, AI‐based models, utilizing NLP, facial recognition, and sensor‐based inputs, offer continuous and objective evaluations [66]. The results of this review are consistent with earlier research, such as the findings by Ray and colleagues, which demonstrated that AI‐powered tools could identify depressive symptoms with a higher accuracy than clinician‐led interviews [67]. Similarly, Montag and colleagues found that AI algorithms using multimodal data sources could predict depressive episodes with high precision, reinforcing AI's growing clinical utility [68]. Moreover, AI's ability to detect subtle emotional cues through speech patterns, typing behavior, and biometric signals provides a significant advantage in identifying mental health deterioration before it becomes clinically visible [69]. Studies by Andrew and colleagues and Zucchetti and colleagues further support the use of real‐time monitoring tools to detect changes in mental states, underscoring the shift from reactive to preventative mental health care, a crucial development for high‐risk populations [70, 71].

Equally significant is the adaptability of AI across different platforms and populations. The reviewed literature illustrates the successful deployment of AI‐powered applications in both clinical and nonclinical settings, ranging from tele‐mental health to mHealth tools. These digital platforms serve as an accessible gateway for individuals who may otherwise avoid traditional mental health services due to stigma or logistical challenges [72]. For example, AI chatbots embedded in mobile applications were found to be particularly appealing to younger users, offering anonymity and immediate, around‐the‐clock support whenever needed [73]. Research by Antoniou and colleagues supports this trend, showing that users often felt more comfortable discussing sensitive topics with AI tools than with human therapists [74]. Likewise, Vial and Almon emphasized that digital mental health interventions powered by AI are effective in engaging hard‐to‐reach populations and reducing care avoidance [75]. Furthermore, studies conducted in rural and low‐resource communities have shown that offline‐capable mental health apps can significantly improve self‐reported well‐being [76, 77]. This affirms the potential of AI to bridge the mental health service gap in underserved areas, offering accessible and cost‐effective solutions that do not depend on a constant professional presence [78]. These outcomes are echoed by Kleine and colleagues, who found that digital mental health platforms successfully expanded access to care in low‐ and middle‐income countries [79].

Beyond individual mental health support, the integration of AI in educational settings demonstrates its value in promoting mental health literacy and institutional responsiveness. AI‐powered educational modules are increasingly being used to teach students about emotional regulation, peer support, and mindfulness, offering interactive and adaptive content tailored to the learner's emotional state [80]. These tools have shown success in improving help‐seeking behavior and reducing stigma, particularly among adolescents and young adults [81]. A study by Golden and colleagues confirmed that AI‐based learning modules improved mental health awareness and emotional resilience in student populations [82]. In addition, AI‐enabled monitoring dashboards used by schools and universities allow for real‐time tracking of student well‐being by analyzing academic and behavioral data [83]. When signs of emotional distress are detected, such as sudden drops in participation or performance, these systems trigger automated check‐ins and alert counseling staff [84]. This proactive model, in alignment with the recommendations of Cabrera and colleagues, facilitates early intervention and reduces the likelihood of crises [85]. This approach aligns with a broader shift toward embedding mental health strategies within institutional frameworks rather than addressing them as isolated services, a concept emphasized in earlier research [86].

Despite these advances, the success of AI‐driven mental health tools is closely tied to user experience and perception. The review highlights that younger users tend to be more receptive to AI tools, appreciating the immediacy, privacy, and flexibility they offer. However, older users, educators, and healthcare providers expressed concerns regarding the impersonality of AI interactions and potential errors in interpreting complex emotional contexts [87]. These concerns are supported by studies such as those by Sinha and colleagues, which emphasize the limitations of AI in delivering empathetic communication, a cornerstone of therapeutic relationships [88]. Technical challenges, such as complex interfaces and unclear usage instructions, were also cited as barriers to engagement [89]. On the other hand, factors that facilitated continued use included ease of navigation, customization options, and clearly communicated data policies [90]. A study by Wang and colleagues found that user‐friendly design and control over personal data significantly influenced ongoing engagement with digital mental health platforms [91]. Notably, privacy remains a dominant concern, particularly around passive data collection from sensors or digital behavior [92]. These findings indicate that developers must prioritize transparency and user autonomy, especially regarding data control, to foster trust and improve long‐term engagement with these technologies [93].

Furthermore, ethical considerations such as data security, algorithmic bias, and explainability remain critical for the responsible deployment of AI in mental health contexts. Although most systems incorporate standard safeguards, such as encryption, anonymization, and user authentication, the potential for breaches or misuse still creates hesitation among users [94, 95]. This aligns with concerns raised in studies by Jain and colleagues, which highlights how unclear privacy policies can erode public trust [96]. Similarly, Rebelo and colleagues stressed the importance of ethical frameworks to guide the deployment of AI in sensitive domains like mental health [97]. Moreover, AI systems trained on limited data sets often perform inconsistently across diverse populations, leading to concerns about fairness and equity [98]. Emotion detection models, for instance, may misinterpret signals from individuals whose communication styles or cultural norms differ from those used in the training data [99]. This issue is reflected in Wang, who found that algorithmic bias in healthcare systems can exacerbate disparities if not addressed through diverse training inputs [100]. As a result, there is a growing push for XAI, which enables both users and professionals to understand how decisions are made [101]. Systems offering visual dashboards, trend analyses, and contextual explanations for alerts tend to enjoy greater user acceptance and reduce resistance from clinicians [102]. Moving forward, AI implementation in mental health should prioritize inclusive data practices, transparent design, and collaborative oversight to ensure that technological progress translates into ethical and equitable outcomes for all users [103].

## 5 Implications for Practice, Policy, and Education

The integration of AI into mental health monitoring has important implications for clinical, policy, and educational domains. Clinically, AI can enhance early detection, personalize treatment, and reduce burden on providers through real‐time emotional monitoring. It also helps standardize assessments and support data‐informed decisions. From a policy perspective, our findings highlight the need for regulatory frameworks that prioritize data privacy, transparency, and inclusive training data sets to prevent bias. Policies should also promote interoperability and incentivize AI use. In education, AI enables discreet well‐being monitoring and early detection of student distress, helping reduce dropout risks. Moreover, integrating AI literacy into mental health curricula can prepare future professionals to responsibly engage with these tools.

Looking ahead, the convergence of AI with immersive digital environments, such as the Metaverse, AR, and VR, presents a promising frontier for mental health support and education [104]. Integrating AI into these platforms could enable dynamic, avatar‐mediated counseling spaces or virtual classrooms tailored to mental health literacy [105, 106]. These technologies may be particularly effective in engaging youth, neurodiverse individuals, and digitally native populations, and merit further exploration as the field evolves [107]. Overall, the implications extend beyond technical feasibility, demanding interdisciplinary collaboration among technologists, clinicians, educators, and policymakers to ensure that AI not only complements but also elevates the standards of mental health care and education in a socially responsible manner.

## 6 Strengths and Limitations

This umbrella review offers several strengths that enhance its relevance and rigor. By synthesizing findings from systematic reviews, scoping reviews, and meta‐analyses, it provides a comprehensive overview of AI applications in mental health monitoring. Use of PRISMA 2020 guidelines and the JBI checklist ensured methodological transparency and reliability. A multidisciplinary search strategy across health and technology databases allowed the inclusion of diverse innovations across clinical and educational settings. Thematic synthesis helped distill complex data into coherent themes relevant to researchers and policymakers.

However, limitations must be noted. Including only English‐language publications may have introduced language bias. The quality and scope of included reviews varied, with some lacking detail on AI models or outcome measures. Given the rapid pace of AI development, some studies may already be outdated. Variability in AI definitions, methods, and evaluation criteria also posed synthesis challenges. As with all umbrella reviews, findings rely on the quality of included sources. Nonetheless, this review contributes valuable insights for future research and practice.

## 7 Conclusion

This umbrella review highlights the growing role of AI in transforming mental health monitoring. AI demonstrated clear utility in early detection of distress, improving diagnostic accuracy, and enabling personalized care through real‐time monitoring. Tools such as wearable devices, ML models, NLP, and chatbots are reshaping mental health support across clinical and nonclinical settings. Notably, several studies emphasized AI's potential to reach underserved and stigmatized populations by overcoming traditional access barriers. However, challenges remain. The findings reflect variability in methodological quality, implementation strategies, and outcome measures. Concerns around algorithmic bias, ethics, and data security also persist. Furthermore, the heterogeneity of AI systems and the lack of standard evaluation frameworks hinder comparative analysis and practical application. Despite these limitations, the evidence supports the responsible use of AI as a promising adjunct to existing mental health services. Interdisciplinary collaboration and ethical governance will be essential to maximizing AI's global impact in this field.

## Author Contributions

**Sumaiya Yeasmin:** conceptualization, investigation, writing – original draft, writing – review and editing, visualization, methodology, formal analysis, resources, data curation. **Mst Masuma Akter Semi:** conceptualization, investigation, writing – review and editing, visualization, validation, formal analysis, data curation, resources, methodology, project administration. **Moustaq Karim Khan Rony:** conceptualization, writing – review and editing, writing – original draft, methodology, software, supervision, formal analysis. **Srabani Das:** conceptualization, investigation, software, data curation, formal analysis, validation, visualization. **Anseena Anees Sabeena:** conceptualization, investigation, writing – review and editing, data curation, formal analysis, methodology, resources. **Rukshanda Rahman:** writing – review and editing, project administration, validation, investigation, conceptualization, data curation. **Barna Biswas:** data curation, writing – original draft, conceptualization, investigation, formal analysis, visualization, project administration, resources. **Fahad Ahmed:** data curation, visualization, conceptualization, methodology, investigation, resources, writing – review and editing, formal analysis. **Adib Hossain:** data curation, resources, writing – review and editing, visualization, project administration, investigation, conceptualization, supervision, formal analysis. All authors have read and approved the final version of the manuscript.

## Funding

The authors received no specific funding for this work.

## Disclosure

The lead author Moustaq Karim Khan Rony affirms that this manuscript is an honest, accurate, and transparent account of the study being reported; that no important aspects of the study have been omitted; and that any discrepancies from the study as planned (and, if relevant, registered) have been explained.

## Ethics Statement

This study did not require an ethical board approval because it did not contain human or animal trials.

## Consent

Informed consent was obtained from all individual participants surveyed in the study.

## Conflicts of Interest

The authors declare no conflicts of interest.

## Data Availability

Data sharing is not applicable to this article as no new data were created or analyzed in this study.
